# The Effect of *Walterinnesia aegyptia* Venom Proteins on TCA Cycle Activity and Mitochondrial NAD^+^-Redox State in Cultured Human Fibroblasts

**DOI:** 10.1155/2015/738147

**Published:** 2015-02-01

**Authors:** Hazem K. Ghneim, Yazeed A. Al-Sheikh, Mourad A. M. Aboul-Soud

**Affiliations:** ^1^Medical and Molecular Genetics Research Chair, Department of Clinical Laboratory Sciences, College of Applied Medical Sciences, King Saud University, P.O. Box 10219, Riyadh 11433, Saudi Arabia; ^2^The Biochemistry Department, Faculty of Agriculture, Cairo University, Giza 12613, Egypt

## Abstract

Fibroblast cultures were used to study the effects of crude *Walterinnesia aegyptia* venom and its F1–F7 protein fractions on TCA cycle enzyme activities and mitochondrial NAD-redox state. Confluent cells were incubated with 10 *μ*g of venom proteins for 4 hours at 37°C. The activities of all studied TCA enzymes and the non-TCA mitochondrial NADP^+^-dependent isocitrate dehydrogenase underwent significant reductions of similar magnitude (50–60% of control activity) upon incubation of cells with the crude venom and fractions F4, F5, and F7 and 60–70% for fractions F3 and F6. In addition, the crude and fractions F3–F7 venom proteins caused a drop in mitochondrial NAD^+^ and NADP^+^ levels equivalent to around 25% of control values. Whereas the crude and fractions F4, F5, and F7 venom proteins caused similar magnitude drops in NADH and NADPH (around 55% of control levels), fractions F3 and F6 caused a more drastic drop (60–70% of control levels) of both reduced coenzymes. Results indicate that the effects of venom proteins could be directed at the mitochondrial level and/or the rates of NAD^+^ and NADP^+^ biosynthesis.

## 1. Introduction

Snake venoms are complex mixtures of protein and nonprotein components [1, 2]. Snake venom proteins are enzymes, toxins, or nerve growth factors. The most important enzymes include phospholipase A_2_, amino acid oxidase, phosphodiesterase, acetylcholinesterase, arginine ester hydrolase, and proteolytic enzymes [3]. Snake venom toxins are proteins that can cause disruption of vital functions. The distinction between enzymes and toxins depends on the fact that many snake venom toxins have no enzyme activity and many enzymes do not have lethal activity. Nerve growth factors are agents that cause differentiation of sympathetic or sensory neurons [4]. The nonprotein fraction of snake venoms includes sodium, potassium, phosphorus, chloride, zinc, magnesium, copper, and manganese. This fraction also contains riboflavin, nucleosides, peptides, amides, lipids, and carbohydrates [5]. The clinical effects of venom proteins include neurotoxic ones causing sensory, motor, cardiac, and respiratory difficulties. Cytotoxic effects are observed on erythrocytes, blood vessels, cardiac muscle, kidneys, lungs, and defects in coagulation and blood cell counts [6]. Effects are also seen consequent to the local release of substances by enzymatic action [7]. The clinical manifestations of envenomation include local swelling, pain, and inflammation [8]. Bleeding occurs but is usually mild and does not require transfusion [9]. Renal and respiratory failure has been shown to occur leading to death [10].

*Walterinnesia aegyptia* (*Wa*, the black desert cobra) is a highly shiny black snake species that belongs to the family Elapidae [11]. It inhabits arid deserts and scrublands and is mainly subterranean with nocturnal activity. The desert cobra is one of the most venomous snakes in Saudi Arabia and is characterized by having rigid, grooved fangs at the front of the upper jaw [12]. Its venom has been shown to be highly neurotoxic, haemorrhagic, and lethal with low proteolytic activity, no necrotic activity, and no effect on blood coagulation or defibrinogenation [13]. Like other elapid snakes, envenomation causes muscular paralysis and respiratory failure in mice and humans [14–17]. Being highly neurotoxic, its venom was previously shown to contain two neurotoxic three-finger toxins that were purified and sequenced [15, 17]. More recently however, three short chain neurotoxic three-finger toxins, two acidic phospholipases, and four Kunitz type protease inhibitors were purified and sequenced from the venom of a single specimen of* Wa* caught in northern Egypt [18]. Being the nonenzymatic markers of elapid venom, the three-finger toxins were shown to display many functions including antagonism of various subtypes of nicotinic acetylcholine receptors, cytotoxicity, cardiotoxicity, inhibition of l-type calcium channels, and acetylcholinesterase [19, 20]. In addition,* Wa* venom has been shown to contain very high activity of various enzymes including phospholipase A_2_ (PLA_2_), l-amino acid oxidase, phosphodiesterase, relatively high hyaluronidase activity, and low proteolytic enzyme activity [13, 21].

Data regarding the effect of venom proteins on cellular and mitochondrial metabolism is very scarce. We have studied the effect of crude and purified venom protein fractions of* Cerastes cerastes gasperetti* (Arabian horned viper),* Echis coloratus* (painted saw-scaled viper), and* Walterinnesia aegyptia* (desert black snake or desert cobra) on the activities of key enzymes related to glucose, glycogen, and amino acid catabolism [22–25] and those of* Cerastes vipera* on mitochondrial respiratory chain activity [26]. In such studies, aliquots of the crude venoms and their protein fractions were incubated with human skin fibroblast cultures as an* in vitro* maintained tissue. In the current study, the same tissue culture model is used to investigate the effect of both crude and purified* Wa* venom protein fractions on mitochondrial TCA cycle activity and the NAD-redox state.

## 2. Materials and Methods

### 2.1. Acquisition of Animals and Preparation of Venom Protein Fractions

Adult* Wa* snakes were collected from the arid areas of Arwa (300 km west of Riyadh, Saudi Arabia). The snakes were kept in the Laboratory of Reptiles, Zoology Department, the College of Science, King Saud University, and maintained in large tanks containing sandy substrata. The present study was approved by the Animal Ethics Committee of College of Science, King Saud University (CS AEC-KSU). Crude venom was milked, purified, and fractionated into seven fractions (F1–F7) as previously described and documented by Al-Saleh et al. [24, 27]. Venom from four desert cobras (120 mg protein/400 *μ*L of venom fluid) was dialyzed overnight in 50 mM acetate buffer (pH 5.8) containing sodium chloride. The venom was centrifuged at 5000 g for 15 min and diluted to 82.5 mg/mL protein. An aliquot (200 *μ*L) was loaded on a TSK G 3000 SW gel filtration column (8 × 300 mm). Elution was performed using the same buffer on an FPLC system (LKB). Protein detection was performed by monitoring absorbance at 280 nm. Fractions 1, 2, 3, 4, 6, and 7 appeared as single bands on both reduced and nonreduced SDS-polyacrylamide gel electrophoresis. The molecular weights of fractions 1, 2, 3, 4, 6, and 7 under nonreducing conditions were 13.5, 65, 70, 16.5, 24.5, and 26.5 kDa, respectively [24, 27]. Fraction 5 showed two bands with molecular weights 23.5 and 12.5 kDa [24, 27]. All of the above experimental work including animal acquisition and preparation of venom protein fractions was performed by Professor Al-Saleh and coworkers [24, 27] and was provided to us as a gift.

### 2.2. Preparation of Human Fibroblast Cultures

The procedure of taking and processing human forearm skin biopsies was approved by the Ethics Committee/IRB of College of Medicine, King Khalid University Hospital, King Saud University (CM IRB-KKUH-KSU). Primary human fibroblast cultures were established from ten forearm skin biopsies (about 20 mg weight) obtained from normal adult volunteers (average age of 27.3 ± 2.4 years). All participating volunteers provided written consent forms. Fibroblasts were cultivated in Eagle's Minimum Essential Medium as described by us previously and confluent cells were subcultured or harvested by trypsinization [22, 23, 25, 26, 28]. Routine growth medium contained Eagle's Minimum Essential Medium (50 mL, 10x concentrated), sterile water (450 mL), 2.2 mM sodium bicarbonate, 10 mM HEPES buffer (pH 7.4), fetal calf serum (10% v/v), penicillin (100 units/mL), streptomycin (0.1 mg/mL), and 2.2 mM l-glutamine. The trypsinization medium contained magnesium and calcium-free Hanks buffered salt solution (50 mL, 10x concentrated), sterile water (450 mL), and 10 mM HEPES (pH 7.4). Sterile trypsin solution (2.5% w/v) was diluted 1 : 10 with trypsinization medium before use. Harvesting medium consisted of magnesium and calcium-free Hanks buffered salt solution (50 mL, 10x concentrated), sterile water (450 mL), and 5 mM sodium bicarbonate. Cells at the early passage 5 of subculture (beginning of their proliferative lifespan) were used for investigation. All fibroblast culture reagents and flasks were purchased from Flow Laboratories, Mclean, VA, USA. Cells were normally cultured in 75 cm^2^ flasks in a Gelaire BSB4 laminar flow cabinet at 37°C in an atmosphere containing 18% O_2_ and 5% CO_2_.

### 2.3. Incubation of Crude Venom and Protein Fractions with Fibroblast Monolayers and Sonicates

The crude venom and venom fractions (F1–F7) were dissolved in Hanks buffered salt solution and aliquots containing 10 *μ*g/200 *μ*L were added to duplicate 75 cm^2^ flasks of the ten confluent passage 5 fibroblast cultures and incubated at 37°C for a period of 4 hrs. Control fibroblast cultures not incubated with venom proteins were run in parallel. The cells from each flask were harvested by trypsinization, resuspended in harvesting medium, washed, and centrifuged at 2000 g for 5 minutes. The pellets were then used to prepare mitochondrial fibroblast sonicates (as described below) for the assay of TCA cycle enzymes.

In a separate experiment, mitochondrial sonicates (0.5 mL) obtained from the fibroblast cultures (not treated with the venom proteins) were incubated in duplicate in a water bath with 10 *μ*g of the crude venom and its protein fractions (F1–F7, 200 *μ*L aliquots) at 37°C for 4 hours. Aliquots of each incubation mixture were then assayed for activities of the various TCA cycle enzymes. This was done to determine if the venom or its fractions exert a direct effect on the enzyme proteins. A fibroblast mitochondrial sonicate (not treated with the venom proteins) was also run in parallel.

### 2.4. Preparation of Mitochondrial Fibroblast Pellets and Sonicates

Cell pellets were homogenized in a potter homogenizer (8–10 strokes) in 0.3 M mannitol solution (8 mL) containing 1 mM EDTA and 10 mM HEPES pH 7.2. The homogenates were then centrifuged at 2000 rpm. Supernatants were kept and the unclear debris was resuspended in mannitol (4 mL) and centrifuged as indicated above. The supernatants (4 + 8 mL) were combined and centrifuged at 10,000 rpm and the mitochondrial pellets collected. Such pellets required the pooling of harvested fibroblasts from fifteen 75 cm^2^ culture flasks. The mitochondrial pellets were suspended in 0.1 mM phosphate buffer pH 7.0 (1 mL), vortexed, and sonicated for 20 seconds in ice using a Fisher Sonic Dismembrator Model 150 at a frequency of 10,000 Hz. Aliquots of the sonicates were then used to assay the activities of the various TCA cycle enzymes as described below. Part of the mitochondrial pellets was treated separately and used to determine the levels of nicotinamide coenzymes as described later.

### 2.5. Enzyme Assays

Citrate synthase (CS) activity was measured by the method of Srere et al. [29]. Mitochondrial sonicate (50 *μ*L) was incubated in the presence of 15 mM Tris buffer pH 8.1 (2 mL), 0.7 mM acetyl-CoA (100 *μ*L), 10 mM dithionitrobenzoic acid (50 *μ*L), and water (700 *μ*L) at 30°C. After a stable baseline signal was obtained, the reaction was initiated by the addition of 0.1 M oxaloacetate (50 *μ*L) and absorbance changes were monitored at 412 nm.

Aconitase (ACN) was assayed according to Hausladen and Fridovich [30]. Mitochondrial sonicate (100 *μ*L) was transferred to a reaction mixture containing 50 mM Tris-HCl, 0.6 mM manganese chloride, 30 mM sodium citrate, and 2 U/mL isocitrate dehydrogenase (NADP^+^-dependant) and the mixture incubated at 37°C. The reaction was started by the addition of 0.2 mM NADP^+^ and absorbance monitored at 340 nm. NAD^+^-dependent isocitrate dehydrogenase (NAD^+^-ICD) and mitochondrial NADP^+^-dependent isocitrate dehydrogenase (NADP^+^-ICD) activities were assayed spectrophotometrically by a modification of the method described by Goncalves et al. [31]. The assay was performed at 25°C in a reaction mixture (1 mL) containing fibroblast sonicate (50 *μ*L), 10 mM KH_2_PO_4_ buffer (pH 7.2), 2 mM MgCl_2_, 2 mM dithiothreitol, 100 *μ*M EDTA, 0.1% Triton X-100 (w/v), and 0.8 mM NAD^+^ (in case of NAD^+^-ICD) or 0.8 m mol/L NADP^+^ (in case of NADP^+^-ICD). Reactions were started by the addition of 15 mM isocitrate, and NAD^+^ or NADP^+^ reduction was monitored at 340 nm and 380 nm, respectively. Activity of *α*-ketoglutarate dehydrogenase (*α*-KGDH) was assayed essentially as described by Lai and Cooper [32]. Mitochondrial sonicate (75 *μ*L) was added to a reaction mixture containing 0.2 mM thiamine pyrophosphate, 2 mM NAD^+^, 1 mM magnesium chloride, 0.4 mM ADP, 10 *μ*M rotenone (an inhibitor of complex I; NADH/ubiquinone oxidoreductase of the respiratory chain), 50 mM potassium phosphate buffer (pH 7.4), and 0.2 mM EGTA. The reaction was initiated by the addition of 0.12 mM coenzyme A and 1 mM *α*-ketoglutarate. Changes in absorbance were monitored at 340 nm and results were calculated using *E*_mM_ = 6.22 for NADH. Succinate dehydrogenase (SDH) was measured as documented by Tan et al. [33]. Mitochondrial sonicate (50 *μ*L) was added to an assay mixture containing 60 *μ*M 2,3-dimethoxy-5-methyl-6-decyl-1,4-benzo-quinone, 50 *μ*M 2,6-dichlorophenolindophenol (terminal electron acceptor), 2 *μ*M rotenone, 5 mM KCN, 1 mM EGTA, 250 mM sucrose, and 50 mM potassium phosphate buffer, pH 7.6 at 37°C. After preincubation for 5 min, the reaction was started by the addition of 20 mM succinate. Absorbance changes were recorded at 600 nm and enzyme activities were calculated using *E*_mM_ = 19.1 for 2,6-dichlorophenolindophenol. Malate dehydrogenase (MDH) activity was measured as described by Kitto [34]. Mitochondrial sonicate (20 *μ*L) was transferred into a reaction mixture containing 10 *μ*M rotenone, 0.15 mM NADH, and 100 mM potassium phosphate buffer, pH 7.4 at 37°C. The reaction was started by the addition of 0.33 mM oxaloacetate and absorbance monitored at 340 nm as described earlier.

Total protein content of mitochondrial sonicates (20 *μ*L) was assayed according to Bradford [35], and enzyme specific activities were expressed as *μ*mole or nmole/min/mg protein. Analysis of variance followed by post hoc Dunnett's *t*-test was performed to evaluate statistical differences between mean ± SD values of enzyme activities and the levels of nicotinamide coenzymes using the SPSS version 17.0 software. Multiple comparisons between sets of data obtained for cells incubated with different venom protein fractions were performed. A *P* < 0.05 value was considered statistically significant.

### 2.6. Measurement of Nicotinamide Coenzymes in Mitochondrial Pellets

Mitochondrial pellets obtained from control fibroblast cultures and those incubated with the crude venom and its protein fractions were used to determine the levels of oxidized (NAD^+^, NADP^+^) and reduced (NADH and NADPH) nicotinamide coenzymes. Mitochondrial pellets were homogenized in 0.1 M HCl (for NAD^+^ and NADP^+^) or in 0.1 M NaOH (for NADH and NADPH) and immediately cooled in an ice bath. The pH of the homogenates was then adjusted to 6.5 with NaOH (for NAD^+^ and NADP^+^) or 7.5 with HCl (for NADH and NADPH) and 0.2 M glycylglycine pH 6.5 or 7.5 (0.5 mL) was added to the oxidized or reduced coenzyme fractions, respectively. Each fraction was then centrifuged (10,000 ×g for 20 minutes at 4°C) to remove insoluble material and supernatants were used for analysis according to documented methodologies [36–38]. For NAD^+^ and NADH measurement, mitochondrial supernatant (20 *μ*g protein) was transferred to an assay mixture containing 50 mM glycylglycine and 0.5 mM EDTA (pH 7.4), 1 mM thiazolyl blue, 0.5 mM phenazine ethosulfate, and alcohol dehydrogenase (final concentration 60 *μ*g/mL). The reaction was then started by the addition of 0.6 M ethanol and the absorbance monitored at 570 nm (30°C). External and internal calibrations with known amounts of NAD^+^ were used to quantitate results. For NADP^+^ and NADPH measurement mitochondrial supernatant (50 *μ*g protein) was added to a reaction mixture containing 50 mM glycylglycine and 0.5 mM EDTA (pH 7.4), 1 mM thiazolyl blue, 0.5 mM phenazine ethosulfate, and 5 mM glucose-6-phosphate dehydrogenase (3.5 U/mL), and absorbance was monitored at 570 nm (37°C). External and internal calibrations with known amounts of NADP^+^ were used to calculate results. The levels of nicotinamide coenzymes were expressed as pmoles/mg protein.

## 3. Results

### 3.1. Effect of Venom Proteins on TCA Cycle Enzyme and NADP^+^-ICD Activities

It is evident from Table 1 data that incubation of ten fibroblast cultures with 10 *μ*g of* Wa* crude venom resulted in highly significant lowering of the specific activities of all studied NAD^+^-dependent TCA cycle enzymes. For example, CS and MDH activities were 29.6 ± 2.33 nmoles/min/mg protein and 0.42 ± 0.03 *μ*moles/min/mg protein, respectively, in fibroblast cultures incubated with the crude venom, compared to 63.8 ± 5.12 nmoles/min/mg protein and 0.96 ± 0.08 *μ*moles/min/mg protein recorded for both enzymes, respectively, in control cultures not incubated with the crude venom (*P* < 0.0001). This was also true for the mitochondrial non-TCA cycle related enzyme NADP^+^-ICD, where the specific activity dropped from 3.38 ± 0.35 nmoles/min/mg protein in control cultures to 1.55 ± 0.14 nmoles/min/mg protein in cell cultures incubated with the crude venom. Interestingly, data reveal that all studied enzymes underwent activity reductions of very similar magnitude in cells incubated with the crude venom which amounted to 55–60% of control activity; *P* < 0.0001 for all comparisons.

Table 1 data further indicate that whereas incubation of fibroblast cultures with F1 and F2 venom protein fractions did not significantly alter the activities of any of the studied enzymes, incubation with F4, F5, and F7 resulted in very similar significant reductions (equal to about 50% of control activity). For example, *α*-KGDH activity was lowered to 0.70 ± 0.06, 0.75 ± 0.07, and 0.69 ± 0.06 nmoles/min/mg protein in cells incubated with F4, F5, and F7, respectively, compared to 1.35 ± 0.12 nmoles/min/mg protein noted in control cells; *P* < 0.0001 for all comparisons. Data also indicate that incubation of fibroblast cultures with 10 *μ*g of F3 and F6 resulted in more drastic reductions in the specific activities of all studied enzymes. Such reductions ranged from about 60 to 70% of the enzymes control activities. For example, NAD^+^-ICD activity was reduced to 0.23 ± 0.03 and 0.24 ± 0.03 nmoles/min/mg protein when cells were incubated with F3 and F6, respectively, compared to 0.76 ± 0.07 nmoles/min/mg protein observed in control cells; *P* < 0.0001. Furthermore, such drastic activity reductions were significantly higher compared to those of all studied enzymes in cells incubated with venom fractions F4, F5, and F7. For example, SDH activity was reduced to 1.40 ± 0.13, 1.43 ± 0.14, and 1.36 ± 0.12 nmoles/min/mg protein, when fibroblasts were incubated with F4, F5, and F7 venom proteins, respectively, compared to 1.07 ± 0.11 and 1.09 ± 0.09 nmoles/min/mg protein when the cells were incubated with F3 and F6 venom proteins, respectively; *P* < 0.001 for all comparisons.

In contrast with all the above findings, Table 2 data indicate that incubation of 10 *μ*g of the crude* Wa* venom and all its protein fractions with mitochondrial fibroblast sonicates did not result in any significant alteration in the activities of any of the studied enzymes.

### 3.2. Effect of Venom Proteins on Mitochondrial NAD-Redox State

As seen from Table 3, incubation of fibroblast cultures with 10 *μ*g of crude* Wa* venom resulted in significant lowering of the mitochondrial levels of both the oxidized and reduced nicotinamide coenzymes NAD^+^, NADP^+^, NADH, and NADPH. The levels of NAD^+^ and NADP^+^ in fibroblasts incubated with the crude venom were 201 ± 12.7 and 21.5 ± 1.47 pmoles/mg protein, respectively, compared to 260 ± 12.3 and 29.8 ± 2.08 pmoles/mg protein, respectively, noted in control cells; *P* < 0.0001 for both comparisons. Such reductions equalled about 23% (for NAD^+^) and 28% (for NADP^+^) of control levels. More drastic reductions however were noted in the levels of NADH and NADPH equalling 58% and 52% of the levels seen in control cells. Such levels were 14.6 ± 1.05 and 40.8 ± 2.83 pmoles/mg protein for NADH and NADPH, respectively, in cells incubated with the crude venom, compared to 34.8 ± 2.87 and 84.6 ± 5.92 pmoles/mg protein, respectively, recorded for control fibroblast cultures; *P* < 0.0001 for both comparisons. Concurrent with the above findings, the NAD^+^/NADH and NADP^+^/NADPH ratios were significantly increased from 7.43 ± 0.29 and 0.35 ± 0.02, respectively, in control cells to 13.9 ± 0.51 and 0.54 ± 0.04, respectively, in fibroblasts incubated with the crude venom; *P* < 0.0001 for both comparisons.

Data in Table 3 also indicate that whereas incubation of fibroblast cultured with 10 *μ*g of F1 and F2 venom proteins did not significantly alter the mitochondrial levels of the studied nicotinamide coenzymes, incubation with the F3, F4, F5, F6, and F7 proteins resulted in very similar significant reductions in the levels of NAD^+^ and NADP^+^ equal to about 23% and 27% of control levels, respectively. Such reduced levels, for example, equalled 198 ± 11.2 and 22.0 ± 1.47 pmoles/mg protein for NAD^+^ and NADP^+^, respectively, in cells incubated with F5, compared to 260 ± 12.3 and 29.8 ± 20.8 pmoles/mg protein, respectively, recorded for control cells. Moreover, it was shown that the incubation of cultures with 10 *μ*g of F4, F5, and F7 resulted in very similar reductions in NADH and NADPH levels equal to about 58% and 51% of control levels, respectively (Table 3). Such levels equalled, for example, 14.8 ± 1.10 and 42.0 ± 2.71 pmoles/mg protein for NADH and NADPH, respectively, in fibroblasts incubated with F4, compared with 34.8 ± 2.87 and 84.6 ± 5.92, respectively, in control cells, *P* < 0.0001 for both comparisons, and are equal in magnitude to the reductions seen upon incubation of cells with the crude venom. Closer examination of Table 3 data, however, indicated that the incubation of cultures with 10 *μ*g of F3 and F6 resulted in similar more drastic reductions in the levels of NADH and NADPH equivalent to about 68% and 60% of control levels, respectively. For example, NADH and NADPH levels were reduced to 11.3 ± 0.89 and 34.1 ± 2.42 pmoles/mg protein, respectively, in fibroblasts incubated with F6, compared to 34.8 ± 2.87 and 84.6 ± 5.92 pmoles/mg protein, respectively, noted in control cells; *P* < 0.0001 for both comparisons. Furthermore, such reductions were significantly higher compared to those seen in cells incubated with F4, F5, and F7 proteins. For example, NADH and NADPH levels were 10.9 ± 0.98 and 33.7 ± 2.92 pmoles/mg protein, respectively, in cells incubated with F3, compared to 15.0 ± 1.14 and 42.3 ± 2.47 pmoles/mg protein in cells incubated with F7; *P* < 0.001 for both comparisons. Concurrent with the above findings, the NAD^+^/NADH and NADP^+^/NADPH ratios were significantly increased from 7.43 ± 0.29 and 0.35 ± 0.02, respectively, in control cells to 13.8 ± 0.60, 14.1 ± 0.58, and 13.5 ± 0.61 (for NAD^+^/NADH) and 0.51 ± 0.03, 0.50 ± 0.05, and 0.50 ± 0.04 (for NADP^+^/NADPH) in cells incubated with F4, F5, and F7, respectively; *P* < 0.0001 for all comparisons. Larger increases in both ratios, however, were obtained when cells were incubated with F3 and F6. Such increases equaled 17.9 ± 0.90 and 17.3 ± 0.76 (for NAD^+^/NADH) and 0.66 ± 0.05 and 0.65 ± 0.05 (for NADP^+^/NADPH) when cells were incubated with F3 and F6, respectively. These ratios were significantly higher than the corresponding ratios obtained for cells incubated with F4, F5, and F7; *P* < 0.001 for all comparisons.

## 4. Discussion

The use of cultured skin fibroblasts as a model of* in vitro* maintained human tissue in this study is justified. We have extensively used this model system to study age-related [28, 39] and venom-related [22–25] changes in intracellular and intramitochondrial metabolism. Optimally cultured human skin fibroblasts are considered rich and versatile in metabolic activity, and results obtained can be extrapolated to hepatocytes. In this study, the system offered the possibility of investigating the effects of different concentrations of the* Wa* venom protein(s) on TCA cycle enzyme activities at increasing incubation times. Preliminary data obtained showed that incubation of fibroblast cultures with 5–50 *μ*g of the venom protein(s) per culture flask resulted in reduction of similar magnitude in the specific activities of all the studied enzymes. Furthermore, maximal effects were observed after 3 hrs of incubation of confluent cells with 10 *μ*g of venom protein fractions 1, 2, 4, and 6. Hence, to minimize kinetic errors, fibroblast cultures were incubated with 10 *μ*g of the venom protein(s) for 4 hrs throughout the study. In addition, fibroblast subcultures between passages 1 and 10 were used for all investigations as cells beyond passage 15 become senescent and have been shown to exhibit very significant variations in the activities of key cytosolic and mitochondrial enzymes [28, 39]. Other optimal culture conditions were provided to allow fibroblasts to grow, multiply, and maximally metabolize. These included the use of sufficient culture medium per flask of cells which was frequently changed, addition of HEPES buffer pH (7.4) in both trypsinization and culture media, and inhibition of bacterial contamination by using streptomycin and penicillin.

It is evident from the results of this study that incubation of confluent fibroblast cultures with both* Wa* dialyzed crude venom and all its protein fractions except F1 and F2 had pronounced significant lowering effects on the specific activities of all studied TCA cycle enzymes plus mitochondrial non-TCA cycle related NADP^+^-specific isocitrate dehydrogenase (Table 1). Very similar reductions in magnitude for all enzyme activities were obtained when the results were expressed in terms of mitochondrial DNA rather than protein (data not shown). The ratio of mitochondrial protein to DNA remained constant regardless of venom incubation (mean = 16.9 *μ*g protein/*μ*g DNA ± 1.53 SD for all cultures). Incubation of the cells monolayer (in 75 cm^2^ flask) with 10 *μ*g venom protein(s) did not change the total protein yield which always came to around 700 *μ*g/flask of cells indicating no proteolytic effect of the venom protein(s). Although proteolytic activity has been associated with some venom proteins [40], that of* Wa* was shown to be low [13]. Moreover, the fact that incubation of fibroblast sonicates with all the* Wa* fractions in this study did not affect the activity of all enzymes indicates that the effect of proteases on such activities is not significant. To this end, some venoms free from such proteolytic activity have been reported [41]. In addition, the absence of venom proteolytic activity in the present investigation could also be attributed to the fact that cells were cultured in medium containing 10% (v/v) foetal calf serum, where a variety of protease inhibitors may have acted to inhibit, at least partially, the venom proteolytic enzyme activity. Furthermore, none of the currently studied enzyme activities could be detected in any of the venom proteins or in the culture medium before or after incubation of the cells with 10 *μ*g of the venom protein(s). Hence, the possibility of cellular or mitochondrial membrane degradation, due to phospholipase A_2_ venom activity, causing enzyme leakage and a drop in cellular activity is ruled out. In contrast, incubation of fibroblast cultures with a much higher concentration of the venom proteins (200 *μ*g/75 cm^2^ flask) caused rupture and lysis of cells.

Other results of the present study showed that the noted reductions in the activity of all studied enzymes could not be seen when the venom proteins were incubated with freshly obtained serum or fibroblast mitochondrial sonicates (Table 2). Furthermore, the reductions in all enzyme activities were of similar magnitude and closely ranged from 50 to 60% of control activity. Such lowered rates of TCA activity could be due to partial inhibition of glucose uptake by the cells under the influence of the venom protein(s). To this end, we have previously shown that incubation of cultured fibroblasts with 10 *μ*g of* Echis coloratus* [25] and* Wa* venom proteins resulted in very significant reductions in the activity of the glycolytic enzyme phosphofructokinase and concurrent very significant increases in glycogen phosphorylase activity, a key enzyme of glycogenolysis. Alternatively, the lowered rates of TCA activity seen suggest that the venom proteins effects are not directed at the enzyme molecules but could be directed at the cellular or mitochondrial level, and may be of mediated nature. In this context, we have previously shown that the effects noted on citrate synthase and creatine kinase activities in fibroblasts incubated with different concentrations of crude* Cerastes cerastes gasperetti* for different periods of time exhibited saturation kinetics [14, 15]. The optimal concentrations of the venom required to cause half-maximal stimulation of creatine kinase and half-maximal inhibition of citrate synthase activities were 0.22 *μ*g and 1.3 *μ*g, respectively, and were achieved after around 1 hr incubation of the cells with the venom. This saturation kinetics phenomenon preliminarily noted in the present study (data not shown) indicates that the venom protein(s) may execute their effects via a receptor binding site present on the outer surface of either the fibroblast cell membrane or the double mitochondrial membrane. This hypothesis can be further investigated by incubating cell cultures with the venom proteins of* Wa* in the presence of proteolytic receptor blockers. In addition, the levels of intracellular messengers such as cAMP and Ca^2+^ can be monitored.

The total mitochondrial NAD^+^/NADH ratio obtained for control fibroblast cultures in the present study equalled 7.43 ± 0.29 (Table 3), and this clearly falls within the expected literature range. Moreover, Table 3 data show that the total mitochondrial NADH concentration in control fibroblasts (34.8 pmol/mg protein) represents 12% of the total NAD^+^ plus NADH concentrations, which again falls within the expected range. Since free NAD^+^ and NADH can be transported from the cytosol into the mitochondria by shuttle systems such as the malate-aspartate shuttle [42], one shortcoming of the present study is whether the mitochondrial NADH monitored originates in the cytoplasm as a result of catalysis of key oxidative enzymes like glyceraldehyde-3-phosphate dehydrogenase or as a result of mitochondrial oxidation catalyzed by the dehydrogenases of the TCA cycle. However, preliminary data obtained upon incubation of control and crude* Wa* venom-treated fibroblasts in the presence of 0.3 mM sodium iodoacetate (an inhibitor of glyceraldehyde-3-phosphate) and 50 mM 2-deoxyglucose (a competitor of glucose) indicated that 95% of the NADH measured originated in the mitochondria (data not shown).

The NAD^+^/NADH ratio plays an important role in regulating the intracellular and intracompartmental redox state and provides a measure of their metabolic activity and health [43]. Many metabolic enzymes are regulated by the NAD^+^/NADH ratio including cytosolic glyceraldehyde-3-phosphate dehydrogenase and the mitochondrial pyruvate dehydrogenase complex which converts pyruvate to acetyl-CoA the opening substrate of the TCA cycle. To this end, it has been shown that the NAD^+^/NADH ratio value fluctuates in response to changes in metabolism [44–47]. In this context, it can be argued that in cases where the mitochondrial NAD^+^/NADH ratio is ~1.0, the value of the ratio will be controlled by small or reasonable changes in NAD^+^ concentrations and hence NAD^+^ can be regarded as the metabolic regulator. However, if the NAD^+^/NADH ratio is of a very high value (in the hundreds), the regulation of the ratio will be more sensitive to a change in NADH concentration and not in NAD^+^. Results of the present study showed that in ten control fibroblast cultures the mitochondrial NAD^+^/NADH ratio equalled 7.43 ± 0.29 which is reasonably close to 1.0 and suggests that under normal conditions mitochondrial metabolism is controlled by NAD^+^ rather than NADH levels. However, upon incubation of fibroblast cultures with the crude* Wa* venom and its F3–F7 protein fractions, the mitochondrial NAD^+^/NADH ratio increased very significantly and ranged from 13.8 ± 0.60 to 16.4 ± 0.76 (Table 3). This increase could have been a direct result of the decreased mitochondrial NADH production subsequent to the very significant inhibition of all studied TCA cycle NAD^+^-dependent dehydrogenases which amounted to 50–60% of control activity (Table 1). Under such abnormal conditions inflicted by the* Wa* venom proteins and the resultant significantly increased NAD^+^/NADH ratio, it is feasible to suggest that mitochondrial NADH rather than NAD^+^ levels have a more prominent role in regulating the ratio and metabolism. Results also showed that the* Wa* venom proteins caused a 25% drop in NAD^+^ fibroblast mitochondrial levels. This could have further contributed to the lowered dehydrogenase activities but was less pronounced than the decrease seen in NADH levels allowing an overall increase in the NAD^+^/NADH ratio. The drop in mitochondrial NAD^+^ levels could have been an outcome of the effect of* Wa* venom proteins on the rate of NAD^+^ synthesis and should be further investigated. A study is underway in our lab to explore such an effect on the activities of several enzymes of NAD^+^ synthesis including nicotinamide/nicotinate mononucleotide adenyltransferase (NMNAT), nicotinamide phosphoribosyltransferase (NAMPT), and NAD^+^ synthase. Because human cells do not synthesize a nicotinamidase, particular interest is given to the role of the nicotinamide recycling enzyme NAMPT as a regulator of NAD^+^ levels in cells. This enzyme is transcriptionally regulated under various conditions, and its expression has been shown to correlate with NAD^+^ concentrations in cultured cells [48]. The importance of NMNAT also stems from the fact that it is required to complete both the de novo and salvage pathways of human NAD^+^ synthesis. In addition, it has been shown to have three separate isoforms localized in nuclei, Golgi, and mitochondria which indicates that NAD^+^ synthesis is compartmentalized [49].

In addition to NAD^+^, cells generate the phosphorylated form of the dinucleotide NADP^+^ by the action of NAD kinase [50]. NADP is predominantly found in its reduced form NADPH required as a strong reducing agent for driving redox reactions mainly associated with anabolic pathways. Thus, the NADP^+^/NADPH ratio is normally maintained at a low value in both cytosolic and mitochondrial fractions [50]. In the present study, the mitochondrial NADP^+^/NADPH ratio in control fibroblasts equalled 0.35 ± 0.02 against 7.43 ± 0.29 obtained for the NAD^+^/NADH ratio. Upon comparison with control fibroblast cultures, incubation of cells with the crude* Wa* venom and its F3–F7 fractions caused a 25% drop in the mitochondrial NADP^+^ levels, a 50–60% drop in NADPH levels, and a concurrent 50–60% drop in NADP^+^-isocitrate dehydrogenase activity. Subsequently the NADP^+^/NADPH ratio underwent a 50–80% increase of its control value. Since these changes were of similar magnitude to those seen for NAD^+^, NADH, and the NAD^+^/NADH ratio, it is suggested that the effects of* Wa* venom proteins on all studied parameters of this study are executed by the same mechanism(s). To this end, it is intended to investigate the effect of the venom proteins on NAD-kinase activity.

In light of the present findings, it is concluded that crude* Wa* venom and its F3–F7 protein fractions caused very significant reductions in all studied TCA cycle enzyme activities and a very pronounced decrease in mitochondrial NADH levels, leading to a significantly increased NAD^+^/NADH ratio. The* Wa* venom proteins also caused a significant decrease in NADP^+^-isocitrate dehydrogenase activity resulting in a very dramatic drop in NADPH levels and a very dramatic increase in the NADP^+^/NADPH ratio. Since the inhibition of all enzyme activities and the increases in the NAD^+^/NADH and NADP^+^/NADPH rates were of similar magnitude, it is possible that the venom proteins execute their effect at the mitochondrial level. In this context, dissolution of mitochondrial cristae and the appearance of mitochondrial vacuoles in the myocardium of* Wa* venom-injected rats were observed in our laboratory [51]. This could have been executed by the high PLA_2_ activity of the venom [13], thus affecting the functional and structural integrity of the mitochondrial fibroblast membrane. Since fractions F3 and F6 caused the highest increases in both the NAD^+^/NADH and NADP^+^/NADPH ratios, these fractions need to be biochemically characterized. Present findings also indicated that mitochondrial NAD^+^ and NADP^+^ levels underwent significant lowering in the presence of the venom proteins which offers an alternative explanation of the decreased NADH and NADPH levels. To this end, the effect of* Wa* venom proteins especially F3 and F6, on the key enzymes of NAD^+^ and NADP^+^ biosynthesis and salvage, should be further investigated. The activities of such enzymes including NMNAT, NAMPT, NAD^+^ synthase, and NAD^+^ kinase are currently under investigation in our lab.

## Figures and Tables

**Table 1:** The effect of incubation of confluent fibroblast cultures with 10 *μ*g of *Walterinnesia aegyptia* venom proteins on the activities of TCA cycle enzymes and mitochondrial NADP^+^-dependent isocitrate dehydrogenase.

Samples (*n* = 10) Control/venom protein	Enzyme specific activities nmole/min/mg protein or *μ*mole/min/mg protein for MDH						
	CS	ACN	NAD^+^-ICD	*α*-KGDH	SDH	MDH	Mitochondrial NADP^+^-ICD
Control	63.8 ± 5.12	6.95 ± 0.61	0.76 ± 0.07	1.35 ± 0.12	2.45 ± 0.21	0.96 ± 0.08	3.38 ± 0.35
Crude venom	29.6 ± 2.33^*^	3.10 ± 0.28^*^	0.31 ± 0.04^*^	0.51 ± 0.04^*^	1.12 ± 0.11^*^	0.42 ± 0.03^*^	1.55 ± 0.14^*^
F1	65.5 ± 5.81	6.73 ± 0.57	0.81 ± 0.07	1.23 ± 0.12	2.26 ± 0.23	0.89 ± 0.08	3.50 ± 0.37
F2	59.6 ± 5.24	7.12 ± 0.60	0.80 ± 0.08	1.29 ± 0.11	2.41 ± 0.24	0.90 ± 0.09	3.31 ± 0.34
F3	24.3 ± 2.71^∗†^	2.98 ± 0.26^∗†^	0.23 ± 0.03^∗†^	0.41 ± 0.05^∗†^	1.07 ± 0.12^∗†^	0.35 ± 0.04^∗†^	1.32 ± 0.14^∗†^
F4	32.8 ± 2.86^*^	3.41 ± 0.30^*^	0.35 ± 0.04^*^	0.70 ± 0.06^*^	1.40 ± 0.13^*^	0.48 ± 0.05^*^	1.52 ± 0.15^*^
F5	36.8 ± 3.10	4.01 ± 0.35^*^	0.36 ± 0.03^*^	0.75 ± 0.07^*^	1.43 ± 0.14^*^	0.45 ± 0.04^*^	1.61 ± 0.17^*^
F6	24.8 ± 2.11^∗†^	2.97 ± 0.26^∗†^	0.24 ± 0.03^∗†^	0.46 ± 0.05^∗†^	1.09 ± 0.09^∗†^	0.37 ± 0.04^∗†^	1.33 ± 0.13^∗†^
F7	30.6 ± 2.50	3.12 ± 0.29^*^	0.33 ± 0.03^*^	0.69 ± 0.06^*^	1.36 ± 0.12^*^	0.44 ± 0.05^*^	1.51 ± 0.14^*^

The values shown here are mean ± SD determined for mitochondrial sonicates of duplicate flasks of ten cultures. CS = citrate synthase, ACN = aconitase, NAD^+^-ICD = NAD^+^-dependent isocitrate dehydrogenase, *α*-KGDH = *α*-ketoglutarate dehydrogenase, SDH = succinate dehydrogenase, and MDH = malate dehydrogenase. NADP^+^-ICD = NADP^+^-dependent isocitrate dehydrogenase. ^*^Significant at *P* < 0.0001 obtained upon comparison of the specific activities of all enzymes in mitochondrial sonicates of cells incubated with the crude venom and fractions F3–F7 against the respective activities recorded for controls. ^†^ *P* < 0.001 obtained upon comparison of the specific activities of all enzymes in mitochondrial sonicates of cells incubated with F3 and F6 against the respective activities recorded for cells incubated with F4, F6, and F7.

**Table 2:** The effect of incubation of mitochondrial fibroblast sonicate with 10 *μ*g of *Walterinnesia aegyptia* venom proteins on the activities of TCA cycle enzymes and mitochondrial NADP^+^-dependent isocitrate dehydrogenase.

Sonicates and venom fractions Assayed samples (*n* = 10)	Enzyme specific activities nmole/min/mg protein or *μ*mole/min/mg protein for MDH						
	CS	ACN	NAD^+^-ICD	*α*-KGDH	SDH	MDH	Mitochondrial NADP^+^-ICD
Sonicate	62.3 ± 5.28	7.20 ± 0.68	0.82 ± 0.08	1.26 ± 0.11	2.56 ± 0.25	1.03 ± 0.09	3.59 ± 0.41
Sonicate + crude venom	58.5 ± 5.12	7.08 ± 0.65	0.77 ± 0.08	1.19 ± 0.10	2.43 ± 0.23	0.97 ± 0.10	3.45 ± 0.38
Sonicate + F1	59.4 ± 5.23	6.92 ± 0.71	0.75 ± 0.07	1.18 ± 0.11	2.38 ± 0.24	1.07 ± 0.11	3.29 ± 0.36
Sonicate + F2	64.9 ± 5.52	6.78 ± 0.56	0.79 ± 0.08	1.21 ± 0.13	2.58 ± 0.26	0.99 ± 0.11	3.51 ± 0.40
Sonicate + F3	66.4 ± 5.91	7.31 ± 0.74	0.84 ± 0.07	1.29 ± 0.13	2.44 ± 0.22	1.06 ± 0.09	3.36 ± 0.41
Sonicate + F4	57.8 ± 4.95	6.82 ± 0.65	0.81 ± 0.08	1.25 ± 0.12	2.35 ± 0.24	1.04 ± 0.10	3.48 ± 0.43
Sonicate + F5	61.4 ± 4.82	6.94 ± 0.67	0.76 ± 0.07	1.17 ± 0.12	2.49 ± 0.23	1.02 ± 0.08	3.56 ± 0.41
Sonicate + F6	58.7 ± 4.67	6.75 ± 0.58	0.79 ± 0.08	1.30 ± 0.13	2.51 ± 0.26	0.98 ± 0.08	3.50 ± 0.39
Sonicate + F7	63.4 ± 5.08	7.32 ± 0.75	0.85 ± 0.09	1.22 ± 0.12	2.60 ± 0.30	0.97 ± 0.07	3.60 ± 0.44

Values shown here are mean ± SD determined for mitochondrial sonicates of duplicate flasks of ten cultures. CS = citrate synthase, ACN = aconitase, NAD^+^-ICD = NAD^+^-dependent isocitrate dehydrogenase, *α*-KGDH = *α*-ketoglutarate dehydrogenase, SDH = succinate dehydrogenase, MDH = malate dehydrogenase, and NADP^+^-ICD = NADP^+^-dependent isocitrate dehydrogenase. No statistically significant differences between all sets of data were observed.

**Table 3:** The effect of incubation of confluent fibroblast cultures with 10 *μ*g of *Walterinnesia aegyptia* venom proteins on mitochondrial NAD-redox state.

Control/venom protein samples (*n* = 10)	Levels of nicotinamide coenzymes (pmole/mg protein)				Redox state	
	NAD^+^	NADH	NADP^+^	NADPH	NAD^+^/NADH	NADP^+^/NADPH
Control	260 ± 12.3	34.8 ± 2.87	29.8 ± 2.08	84.6 ± 5.92	7.43 ± 0.29	0.35 ± 0.02
Crude venom	201 ± 12.7^*^	14.6 ± 1.05^*^	21.5 ± 1.47^*^	40.8 ± 2.83^*^	13.9 ± 0.51^*^	0.54 ± 0.04^*^
F1	252 ± 13.1	34.9 ± 2.27	28.4 ± 1.92	80.2 ± 5.61	7.36 ± 0.31	0.34 ± 0.02
F2	267 ± 15.1	37.2 ± 2.82	30.7 ± 2.11	82.5 ± 5.66	7.50 ± 0.28	0.36 ± 0.02
F3	199 ± 9.43^*^	10.9 ± 0.98^∗†^	22.8 ± 1.55^*^	33.7 ± 2.92^∗†^	17.9 ± 0.90^∗†^	0.66 ± 0.05^∗†^
F4	203 ± 10.9^*^	14.8 ± 1.10^*^	21.1 ± 1.38^*^	42.0 ± 2.71^*^	13.8 ± 0.60^*^	0.51 ± 0.03^*^
F5	198 ± 11.2^*^	14.1 ± 1.08^*^	22.0 ± 1.47^*^	43.2 ± 2.55^*^	14.1 ± 0.58^*^	0.50 ± 0.05^*^
F6	194 ± 9.80^*^	11.3 ± 0.89^∗†^	21.8 ± 1.30^*^	34.1 ± 2.42^∗†^	17.3 ± 0.76^∗†^	0.65 ± 0.05^∗†^
F7	202 ± 10.6^*^	15.0 ± 1.14^*^	20.9 ± 1.41^*^	42.3 ± 2.47^*^	13.5 ± 0.61^*^	0.50 ± 0.04^*^

The values shown here are mean ± SD determined for mitochondrial sonicates of duplicate flasks of ten cultures. NAD^+^ = oxidized nicotinamide adenine dinucleotide. NADH = reduced nicotinamide adenine dinucleotide. NADP^+^ = oxidized nicotinamide adenine dinucleotide phosphate. NADPH = reduced nicotinamide adenine dinucleotide phosphate.

^*^Significant at *P* < 0.0001 obtained upon comparison of the levels of all studied parameters in mitochondria of cells incubated with fractions F3–F7 against the respective levels recorded for controls.

^†^ *P* < 0.001 obtained upon comparison of the levels of all studied parameters in mitochondria of cells incubated with fractions F3 and F6 against the respective levels recorded for cells incubated with F4, F5, and F7.
